# Interaction between opposing modes of phospho-regulation of the proneural proteins Ascl1 and Ngn2

**DOI:** 10.12688/wellcomeopenres.14848.1

**Published:** 2018-10-01

**Authors:** Laura J.A. Hardwick, Anna Philpott

**Affiliations:** 1 Wellcome-MRC Cambridge Stem Cell Institute, University of Cambridge, Cambridge, CB2 1QR, UK; 2 Department of Oncology, University of Cambridge, Cambridge, CB2 0XZ, UK; 3 Peterhouse, University of Cambridge, Cambridge, CB2 1RD, UK

**Keywords:** Proneural, phospho-regulation, neurogenesis, Ascl1, Neurogenin2, Xenopus.

## Abstract

From the relatively simple nervous system of *Drosophila* to the elaborate mammalian cortex, neurogenesis requires exceptional spatial and temporal precision to co-ordinate progenitor cell proliferation and subsequent differentiation to a diverse range of neurons and glia. A limited number of transiently expressed proneural basic-helix-loop-helix (bHLH) transcription factors, for example *achaete-scute-complex (as-c)* and *atonal (ato)* in *Drosophila* and the vertebrate homologues Ascl1 and Neurogenin2 (Ngn2), are able to orchestrate the onset of neuronal determination, context-dependent subtype selection and even influence later aspects of neuronal migration and maturation. Within the last decade, two models have emerged to explain how the temporal activity of proneural determination factors is regulated by phosphorylation at distinct sites. One model describes how cell-cycle associated phosphorylation on multiple sites in the N and C termini of vertebrate proneural proteins limits neuronal differentiation in cycling progenitor cells. A second model describes phosphorylation on a single site in the bHLH domain of *Drosophila atonal* that acts as a binary switch, where phosphorylation terminates proneural activity. Here we combine activating mutations of phosphorylation sites in the N- and C- termini with an inhibitory phospho-mimetic mutation in the bHLH domain of Ascl1 and Ngn2 proteins, and test their functions *in vivo* using *Xenopus* embryos to determine which mode of phospho-regulation dominates. Enhancing activity by preventing N- and C terminal phosphorylation cannot overcome the inhibitory effect of mimicking phosphorylation of the bHLH domain. Thus we have established a hierarchy between these two modes of proneural protein control and suggest a model of temporal regulation for proneural protein activity.

## Introduction

Development of the nervous system requires elaborate control to expand progenitor cells before subsequent differentiation into a diverse array of neuronal and glial subtypes. One of the most striking features is that a limited number of proneural basic-helix-loop-helix (bHLH) determinant factors, for example *achaete-scute-complex (as-c)* and *atonal (ato)* in *Drosophila* and the vertebrate homologues Ascl1 and Neurogenin2 (Ngn2), are able to orchestrate both generic neuronal determination and context-dependent neuronal subtype selection ^1^. Furthermore, these determination factors are able to influence relatively late aspects of neuronal migration and maturation despite having only a transient window of expression ^1^. Proneural protein activity is exquisitely regulated both spatially and temporally ^2, 3^, and mechanisms are conserved from *Drosophila* neurogenesis to the mammalian cortex.

The mechanisms that underlie the complexity of the developing nervous system have begun to be elucidated. For example there are multiple bi-directional links that intimately connect and influence the transition between progenitor cell proliferation and neuronal differentiation during neurogenesis ^4^. Within the last decade, models of phospho-regulation have emerged to explain the temporal control and/or context-dependent activity of proneural determination factors ^5– 7^. Modification of single regulatory phospho-sites may fine-tune activity of individual proneural proteins within a given context, for example GSK3β-mediated phosphorylation of Ngn2 to promote motor neuron formation ^8^. Additionally, models have been described to regulate generic proneural activity in progenitor cells.

Firstly, a multi-site phospho-regulatory model is described for vertebrate Ascl1 and Ngn2, based on data from *Xenopus* embryos and mammalian cell culture ^5, 6, 9, 10^. Cyclin-dependent-kinase activity that promotes the cell cycle can additionally phosphorylate proneural proteins on multiple Serine-Proline sites located in the N and C termini either side of the basic helix-loop-helix domain that regulates DNA binding and dimerisation. This multi-site phosphorylation limits protein stability and chromatin association, and specifically reduces activation of genes associated with cell cycle exit and differentiation ^5, 6^. Correspondingly, Serine to Alanine phospho-mutant proneural proteins that cannot undergo this phosphorylation promote differentiation during development, and in cellular reprogramming assays ^5, 6, 9^.

Secondly, a single regulatory phospho-site within the bHLH domain is described for *atonal* in *Drosophila* retina and Ngn2 in mouse cortex; while the phospho-mutant versions behave as the wild-type proteins, the respective Serine/Threonine to Aspartic acid phospho-mimetics are inactive ^7^. Phosphorylation of this single site in *atonal* acts a binary switch to terminate proneural activity in retinal R8 precursors ^7^. Here we present a short study to determine the effect of combining the activating mutations blocking phosphorylation of the N and C-termini of mouse Ascl1 and Ngn2 with an inhibitory phospho-mimetic mutation in the bHLH domain, to determine whether one mode of phospho-regulation is dominant over the other.

## Methods

### Animal care

All work has been carried out under UK Home Office Licence and has passed an Institutional ethical review committee assessment at the University of Cambridge.

### Plasmids and constructs

Wild-type mouse *Ascl1* (Genbank accession number NM008553) and 6S-A phospho-mutant mouse *Ascl1* were published in 9. Wild-type mouse Ngn2 (Genbank accession number NM009718) and 9S-A mouse Ngn2 were published in 5. The S150D and T149D substitutions were introduced by Quikchange II site directed mutagenesis (Agilent Technologies). Primers:

mAscl1_S150D_S = CGGCCAACAAGAAGATGGACAAGGTGGAGACGCTGC;

AS = GCAGCGTCTCCACCTTGTCCATCTTCTTGTTGGCCG.

 mNgn2_T149D_S = CCGAGGATGCCAAGCTCGATAAGATCGAGACGCTGCG,

 AS = CGCAGCGTCTCGATCTTATCGAGCTTGGCATCCTCGG.

### *Xenopus laevis* embryo manipulation

All efforts are made to ameliorate suffering to any animal. For example, the colony of approximately 80 *X. laevis* females are housed and cared for by a dedicated team of animal technicians operating under Home Office Licence. Each experiment requires eggs from 2 or 3 females (depending on N = 2 or 3) and females are used on rotation within the colony with at least a 3-month rest period after laying. A single male frog is sacrificed under humane conditions and Home Office Licence to provide testes for at least 16 experiments. Embryos obtained from fertilised eggs are used for the experiments and development is stopped 48 hours post fertilisation when embryos reach late neurula stage and prior to formation of tadpoles.

Thus, *X. laevis* eggs were obtained by standard hormone methods of induction, and subsequently fertilised *in vitro*. pCS2+ constructs were linearised and capped mRNA was transcribed *in vitro* using the SP6 mMessage mMachine ^® ^kit (Ambion). Embryos were injected unilaterally at the two cell stage with mRNA as indicated in the text, with GFP (for qPCR) or β-gal (ISH) as lineage tracers. Embryos were cultured at 18°C in Ficol solution and staged according to 11. At stage 18, embryos were either snap-frozen for qPCR analysis or fixed in MEMFA for 90 minutes, as described in 12.

### Whole mount *in situ* hybridisation (ISH)

Dig-oxigenin-labelled anti-sense probes were synthesised from plasmid *X. laevis neural-β-tubulin* ^13^. Whole mount ISH was performed as described in 12 and embryos were scored for the extent and pattern of gene expression as described in data analysis.

### Quantitative real-time PCR (qPCR)

GFP expression was used to confirm successful injection and samples of four embryos were snap frozen. Whole embryo RNA was extracted using the RNeasy ^®^ Mini kit (Qiagen) and template cDNAs synthesised with the QuantiTect ^®^ Reverse Transcription Kit (Qiagen). qPCR was performed using the Quantifast ^®^ SYBR Green PCR kit (Qiagen) in a LightCycler ^®^ 480 (Roche). Thermal cycling conditions: 95°C for 5 minutes, then 45 cycles of 95°C for 10s, 60°C for 10s and 72°C for 20s. EF1α reference gene (Genbank accession NM001087442): Forward, CACCATGAAGCCCTTACTGAG; Reverse, TGATAACCTGTGCGGTAAATG. N-β-Tubulin target gene (Genbank accession NM001086064): Forward, TGGATTTGGAACCAGGCA; Reverse, GCTCAGCTCCTTCGGTGTA.

### Western blotting

For western blot analysis, 12 embryos were snap frozen at stage 11 and whole embryo protein was extracted as described in 12. 50µg total protein was loaded on to pre-cast BioRad Criterion ^™^ TGX 18% gels in Tris-Glycine buffer. Primary antibodies were used at 1:2000 dilution for at least 1 hour at room temperature (tubulin) or at 4°C overnight (HA): Rat HRP-conjugated anti-HA clone 3F-10 antibody (Roche; 12013819001) and mouse anti-α-tubulin clone B-5-1-2 antibody (Sigma; T5618). Anti-tubulin antibody was detected with a sheep HRP-conjugated anti-mouse antibody at 1:10000 dilution (GE Healthcare; NA931V).

### Data analysis

For ISH data, embryos were scored for the extent and pattern of N-β-Tubulin expression on the injected side relative to uninjected side and to uninjected embryos. Scores were assigned as 0, no difference; +1, mild increase in expression within the neural tube with or without occasional ectopic expression on the injected side; +2, moderate increase with ectopic expression occurring in patches on the injected side and sometimes bilaterally; +3, substantial increase with extensive ectopic expression in a more homogenous pattern on the injected side and sometimes bilaterally. Experiments were conducted in independent duplicate and the N numbers refer to the range of total numbers of embryos in each injection category.

For qPCR data, mRNA expression was normalised to expression of reference gene EF1α and mRNA levels in the injected embryos were calculated relative to stage-matched uninjected controls. Mean values are plotted and error bars show the standard error of the mean from two independent experiments. Statistical significance was calculated by a paired two-tailed student T test in Microsoft Excel 2010; NS = not significant; * = p< 0.05; ** = p< 0.025; *** = p< 0.0125. Western blot is representative from two independent experiments. For protein quantification, Image J software was used as described in 12.

## Results

### Generation of combined phospho-mutant and phospho-mimetic constructs

Experiments supporting the regulatory model based on multi-site phosphorylation in the N- and C-termini of proneural proteins has been well documented in *Xenopus*, mouse and human proneural proteins ^5, 6, 9, 10, 12^, with conservation of the model across germ layers to include bHLH proteins in other tissues such as MyoD in muscle/mesoderm ^14^ and Ngn3 in pancreas/endoderm ^15^. In each case, the regulatory phospho-sites are located in the N and C terminal domains, and preventing phosphorylation by mutating Serine/Threonine-Proline sites to Alanine-Proline results in enhanced protein stability and increased DNA binding affinity. This enables expression of slower responding differentiation genes that tend to be less epigenetically available; reviewed in 16. Hence de-phosphorylation of these sites enhances the ability of bHLH proteins to drive differentiation.

In contrast, phosphorylation of a single site in the bHLH domain of *Drosophila atonal* in the R8 photoreceptors of the *Drosophila* retina terminates proneural activity, and this mode of regulation appears to be conserved with Ngn2 in murine cortical progenitors ^7^. This single conserved site at the junction between the loop region and helix 2 is predicted to lie in the near vicinity of negatively charged phosphates in the DNA backbone; phosphorylation of the site, possibly mediated by Protein Kinase A, reduces DNA binding to inactivate *atonal* upon R8 precursor selection ^7^.

**Figure 1.**
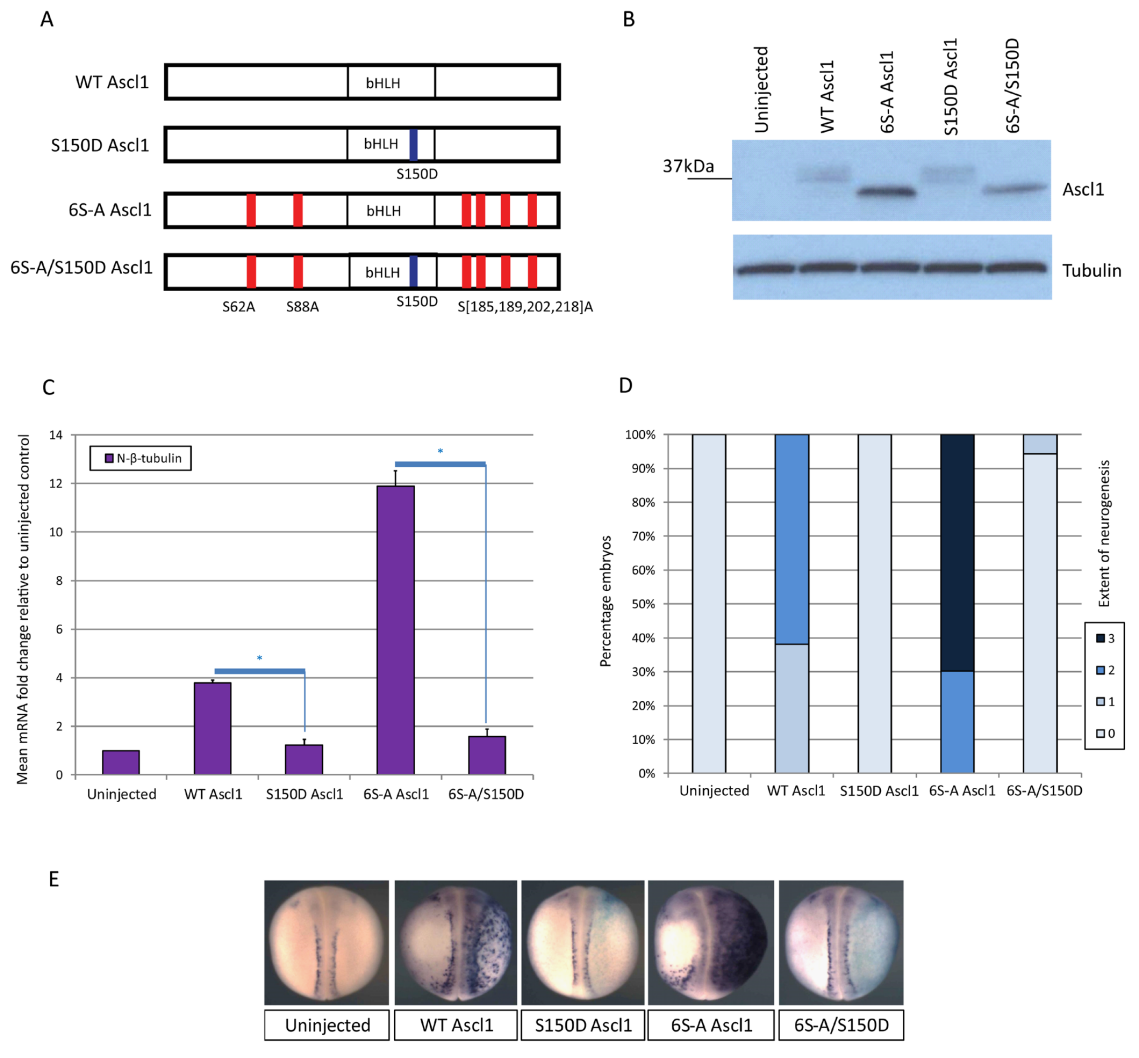
S150D single site mutation inactivates both WT and 6S-A Ascl1. ( **A**) Schematic representation of WT Ascl1 and the three mutant constructs tested. The relative location of the S150D phospho-mimetic substitution within the bHLH domain is indicated (blue), along with the location of the six Serine to Alanine phospho-mutant substitutions in the N and C termini (red). ( **B**) Western blot analysis of stage 11 whole embryo extracts over-expressing 200pg of each construct, with tubulin as a loading control. All four constructs are expressed in embryos and the additional S150D mutation has no significant effect on WT or 6S-A Ascl1 protein migration or accumulation. ( **C**– **E**) Two cell stage embryos were unilaterally injected with 40pg of mRNA encoding each construct. At stage 18, embryos were assayed for expression of neural-β-tubulin relative to uninjected control embryos. ( **C**) qPCR data [N=2] with significance calculated by paired student T test; * = p< 0.05. ( **D**) Semi-quantitative scoring of grade of neurogenesis after ISH [N=53-73 embryos per category from two experiments]. ( **E**) Representative images of embryos with injected side to the right, stained with pale blue β-gal tracer. Induction of neural-β-tubulin by WT and 6S-A Ascl1 is prevented with the respective introduction of the single S150D mutation.

**Figure 2.**
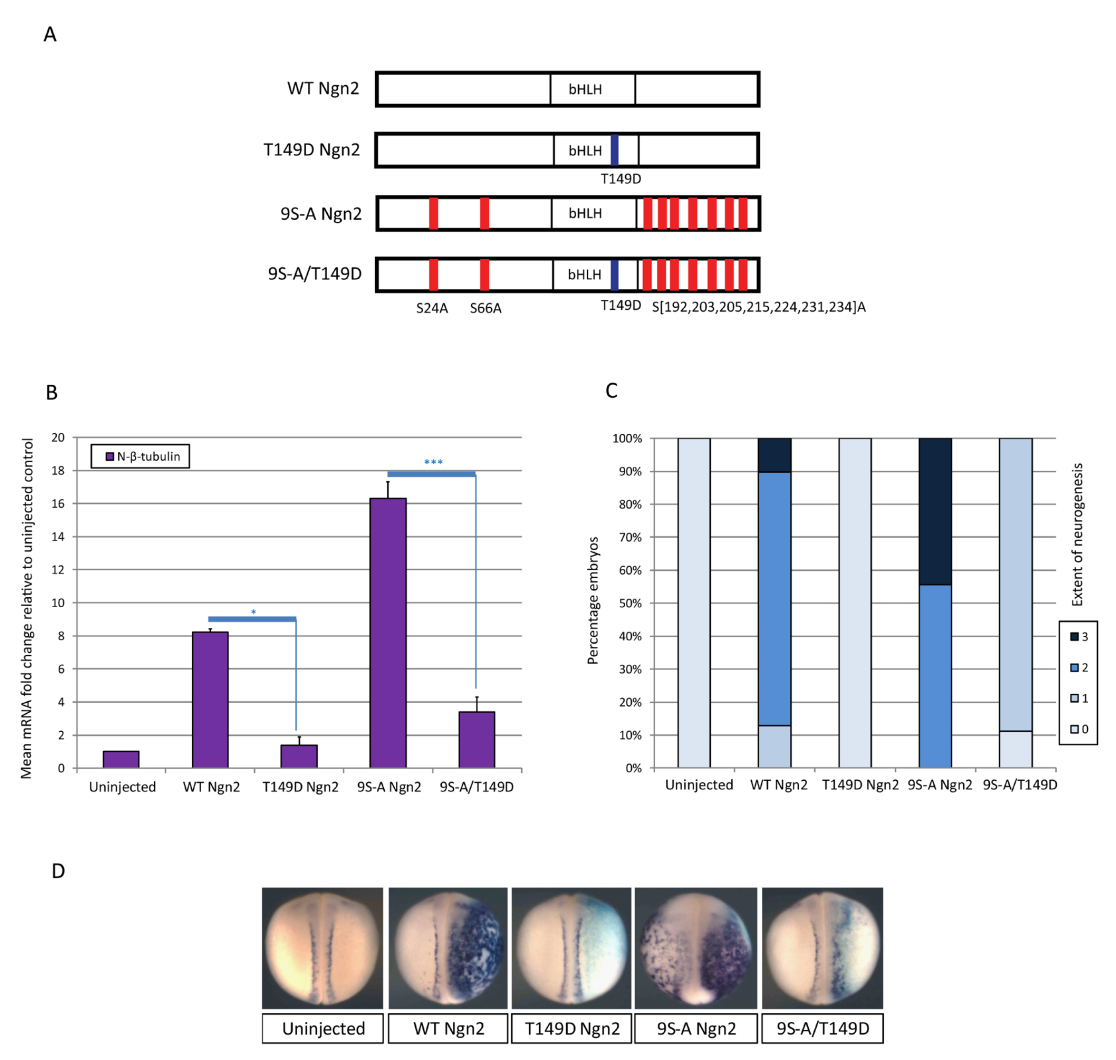
T149D single site mutation significantly inhibits both WT and 9S-A Ngn2. ( **A**) Schematic representation of WT Ngn2 and the three mutant constructs tested. The relative location of the T149D phospho-mimetic substitution within the bHLH domain is indicated (blue), along with the location of the nine Serine to Alanine phospho-mutant substitutions in the N and C termini (red). ( **B**– **D**) Two cell stage embryos were unilaterally injected with 25pg of mRNA encoding each construct. At stage 18, embryos were assayed for expression of neural-β-tubulin relative to uninjected control embryos. ( **B**) qPCR data [N=2] with significance calculated by paired student T test; * = p< 0.05; *** = p< 0.0125. ( **C**) Semi-quantitative scoring of grade of neurogenesis after ISH [N=36–39 embryos per category from two experiments]. ( **D**) Representative images of embryos with injected side to the right, stained with pale blue β-gal tracer. Introduction of the T149D mutation prevents induction of neural-β-tubulin by WT Ngn2 and significantly inhibits 9S-A Ngn2.

The effect of manipulating the single bHLH domain regulatory site has not previously been investigated in mouse Ascl1 protein (homologue of *Drosophila as-c*), nor has the effect of modulation been tested in the *Xenopus* system. Hence, in the present study, the equivalent phospho-mimetic construct was made with substitution of Serine 150 to Aspartic acid (S150D) in wild-type (WT) mAscl1. To explore the effect of combining this potentially inactivating mutation with activating mutations that prevent phosphorylation on multiple N- and C-terminal sites, the same S150D phospho-mimetic substitution was made into 6S-A mAscl1, where six Serine-Proline (SP) sites in the N and C termini have been mutated to Alanine-Proline ( Figure 1A). In addition, a Threonine 149 phospho-mimetic (T149D) form of mNgn2 ^7^ was recreated in both a wild-type and a 9S-A Ngn2 background, where nine Serine-Proline (SP) sites in the N and C termini of mNgn2 have been mutated to Alanine-Proline ^5^ ( Figure 2A). All constructs contain a single C terminal HA tag.

### The single-site bHLH phospho-mimetic within the bHLH domain inactivates Ascl1 activity

Early development of *Xenopus laevis* is an excellent *in vivo* assay system in which to explore proneural protein activity; over-expression of proteins such as Ascl1 and Ngn2 by microinjection of *in vitro* transcribed mRNA results in induced neurogenesis and expression of neural-β-tubulin across the lateral ectoderm ^5, 6^. Initially focusing on Ascl1, to confirm protein expression whole embryo extracts were made from embryos over-expressing equal amounts of mRNA encoding each of the four Ascl1 constructs ( Figure 1A, B). All four constructs are successfully expressed in *Xenopus* embryos: WT Ascl1 and S150D Ascl1 migrate as broad bands while 6S-A Ascl1 and 6S-A/S150D Ascl1 appear as single faster migrating bands, consistent with the former pair being phosphorylated on the sites mutated in the latter pair.

Additionally, 6S-A Ascl1-based proteins accumulate more than the respective WT proteins, consistent with an increase in protein half-life of this phospho-mutant ^6^. While there is a trend towards a reduced protein density in 6S-A/S150D compared to 6S-A Ascl1, when measured relative to the tubulin loading control from repeat experiments, the difference between these is not statistically significant (p=0.078). Thus, the introduction of the S150D phospho-mimetic into the WT or 6S-A Ascl1 constructs does not significantly affect the protein accumulation.

To test for functional effects of these different forms of Ascl1, mRNA encoding each construct was unilaterally injected into two cell stage embryos, and neurogenesis was assayed at stage 18 by qPCR or *in situ* hybridisation (ISH) for expression of N-β-tubulin ( Figure 1C–E), a marker of primary neurogenesis. As previously reported, over-expression of both WT and 6S-A Ascl1 induces ectopic neurogenesis on the injected side both within and outside the neural plate ^6^. In direct comparison, 6S-A Ascl1 shows enhanced proneural activity relative to WT Ascl1, inducing N-β-tubulin expression more extensively over the lateral ectoderm and three-fold higher transcript accumulation by qPCR analysis. In contrast, the introduction of the single S150D phospho-mimetic dramatically inactivates both WT and 6S-A Ascl1, consistent with inhibition of DNA binding as described previously ^7^. If so, this suggests that the superior activity of 6S-A relative to WT Ascl1 requires direct DNA binding.

### The single-site bHLH phospho-mimetic inactivates Ngn2 activity

To complement these experiments with Ascl1, we undertook a comparable analysis, investigating the activity of WT and 9S-A mouse Ngn2 with and without the T149D substitution in the bHLH domain ^5, 7^. Ngn2 is more potent than Ascl1 in upregulating N-β-tubulin in *Xenopus* embryos. Hence, mRNA quantities for injection were reduced to 25pg with assay of N-β-tubulin induction as before ( Figure 2B–D). Both WT and 9S-A Ngn2 induce extensive ectopic expression of N-β-tubulin within and outside the neural plate, and on qPCR analysis, 9S-A Ngn2 induces approximately twice the level of N-β-tubulin transcripts as WT Ngn2. As with Ascl1, the introduction of the single T149D mimetic strikingly inhibits the ability of both WT and 9S-A Ngn2 to induce ectopic neurons. However, in contrast to Ascl1, some residual activity is maintained in 9S-A/T149D Ngn2 but this is reduced to expansion of the neural tube only rather than induction of ectopic neurogenesis.

## Discussion/conclusions

In this study, we firstly demonstrate that introduction of a single phospho-mimetic residue into the bHLH domain of mouse Ascl1 inactivates its ability to drive neurogenesis, as previously described in *Drosophila atonal* ^7^. We next investigated Ascl1 and Ngn2 proneural activity in the presence of activating mutations in the N- and C-termini ^5, 6^, together with inhibitory phospho-mimetic mutation in the bHLH domain ^7^. We find that inhibition of proneural activity by this inhibitory phospho-mimetic mutation is dominant. S/T-D substitutions in the bHLH domain reduce DNA binding of the proneural protein ^7^ so it is reasonable to assume that S-A mutant Ascl1 and Ngn2 require DNA binding for their enhanced activity in *Xenopus,* but we have not tested this hypothesis directly. It would be interesting to determine whether residual activity in the 9S-A/T149D mutant of Ngn2 represents residual DNA binding or acts via a DNA-binding independent mechanism, e.g. by complexing with other DNA-bound factors.

The binary switch model where a single phosphorylation event in the bHLH domain controls DNA binding is an elegant way to achieve a rapid and sharp change in proneural activity in response to the cellular environment that is not dependent on protein degradation or subcellular translocation ^7^. In contrast, multi-site phosphorylation of the N- and C-termini works much more like a rheostat where decreasing levels of Cyclin-dependent-kinase activity at the transition towards differentiation leads to a more gradual change in phospho-status and proneural activity ^5, 6^. Combining both models, a temporal scheme could be suggested where sequential kinase activity exquisitely controls neurogenesis. In actively proliferating neural progenitor cells, proneural activity may be restrained by multi-site phosphorylation of proneural factors by Cyclin/Cyclin-dependent-kinases to enable sufficient progenitor cell expansion. At the transition to differentiation, where the cell cycle slows and lengthens, gradual de-phosphorylation of proneural proteins may tip the balance in favour of differentiation and activation of downstream gene cascades required to drive neurogenesis. Once these cascades are activated, determination proneural factor activity could be rapidly halted by phosphorylation of the bHLH domain, possibly by PKA or other later acting kinases ^7^. In this way, combining these two modes of phospho-regulation in proneural proteins could provide an explanation for temporal regulation of generic proneural determination activity from proliferating progenitor cells through to differentiating neurons.

## Data availability

Raw data files available in Open Science Framework: Interaction between opposing modes of phospho-regulation of the proneural proteins Ascl1 and Ngn2, https://doi.org/10.17605/OSF.IO/73NE5 ^17^

See *Methods* section for description of data analysis and ISH scoring. Datasets presented are as follows:

- Fig1B_WesternBlot with Fig1B_Loading
- Fig1C_qPCR: Mean fold change in N-β-tubulin expression relative to uninjected controls; two independent experiments.
- Fig1D_ISH: Semi-quantitative scoring of extent of neurogenesis for each injection category showing total number of embryos from two independent experiments.
- Fig1E_embryos: Representative images from 53–73 embryos in each category in two independent experiments.
- Fig2B_qPCR: Mean fold change in N-β-tubulin expression relative to uninjected controls; two independent experiments.
- Fig2C_ISH: Semi-quantitative scoring of extent of neurogenesis for each injection category showing total number of embryos from two independent experiments.
- Fig2D_embryos: Representative images from 36–39 embryos in each category in two independent experiments.

Data are available under the terms of the Creative Commons Zero "No rights reserved" data waiver (CC0 1.0 Public domain dedication).

## References

[1] Bertrand N Castro DS Guillemot F: Proneural genes and the specification of neural cell types. *Nat Rev Neurosci.* 2002;3(7):517–30. 10.1038/nrn874 12094208

[2] Baker NE Brown NL: All in the family: proneural bHLH genes and neuronal diversity. *Development.* 2018;145(9): pii: dev159426. 10.1242/dev.159426 29720483 PMC5992591

[3] Wilkinson G Dennis D Schuurmans C: Proneural genes in neocortical development. *Neuroscience.* 2013;253:256–73. 10.1016/j.neuroscience.2013.08.029 23999125

[4] Hardwick LJ Ali FR Azzarelli R: Cell cycle regulation of proliferation versus differentiation in the central nervous system. *Cell Tissue Res.* 2015;359(1):187–200. 10.1007/s00441-014-1895-8 24859217 PMC4284380

[5] Ali F Hindley C McDowell G: Cell cycle-regulated multi-site phosphorylation of Neurogenin 2 coordinates cell cycling with differentiation during neurogenesis. *Development.* 2011;138(19):4267–77. 10.1242/dev.067900 21852393 PMC3171226

[6] Ali FR Cheng K Kirwan P: The phosphorylation status of Ascl1 is a key determinant of neuronal differentiation and maturation *in vivo* and *in vitro*. *Development.* 2014;141(11):2216–24. 10.1242/dev.106377 24821983

[7] Quan XJ Yuan L Tiberi L: Post-translational Control of the Temporal Dynamics of Transcription Factor Activity Regulates Neurogenesis. *Cell.* 2016;164(3):460–75. 10.1016/j.cell.2015.12.048 26824657

[8] Ma YC Song MR Park JP: Regulation of motor neuron specification by phosphorylation of neurogenin 2. *Neuron.* 2008;58(1):65–77. 10.1016/j.neuron.2008.01.037 18400164 PMC2587148

[9] Wylie LA Hardwick LJ Papkovskaia TD: Ascl1 phospho-status regulates neuronal differentiation in a *Xenopus* developmental model of neuroblastoma. *Dis Model Mech.* 2015;8(5):429–41. 10.1242/dmm.018630 25786414 PMC4415893

[10] Hindley C Ali F McDowell G: Post-translational modification of Ngn2 differentially affects transcription of distinct targets to regulate the balance between progenitor maintenance and differentiation. *Development.* 2012;139(10):1718–23. 10.1242/dev.077552 22491944 PMC3328174

[11] Nieuwkoop PD Faber J: Normal table of Xenopus laevis. New York: Garland Publishing;1994 Reference Source

[12] Hardwick LJ Philpott A: Multi-site phosphorylation regulates NeuroD4 activity during primary neurogenesis: a conserved mechanism amongst proneural proteins. *Neural Dev.* 2015;10:15. 10.1186/s13064-015-0044-8 26084567 PMC4494719

[13] Vernon AE Devine C Philpott A: The cdk inhibitor p27 ^Xic1^ is required for differentiation of primary neurones in *Xenopus*. *Development.* 2003;130(1):85–92. 10.1242/dev.00193 12441293

[14] Hardwick LJ Davies JD Philpott A: MyoD phosphorylation on multiple C terminal sites regulates myogenic conversion activity. *Biochem Biophys Res Commun.* 2016;481(1–2):97–103. 10.1016/j.bbrc.2016.11.009 27823936 PMC5127879

[15] Azzarelli R Hurley C Sznurkowska MK: Multi-site Neurogenin3 Phosphorylation Controls Pancreatic Endocrine Differentiation. *Dev Cell.* 2017;41(3):274–86.e5. 10.1016/j.devcel.2017.04.004 28457793 PMC5425251

[16] Philpott A: Multi-site phospho-regulation of proneural transcription factors controls proliferation versus differentiation in development and reprogramming. *Neurogenesis (Austin).* 2015;2(1):e1049733. 10.1080/23262133.2015.1049733 27502783 PMC4973605

[17] Hardwick L: Interaction between Opposing Modes of Phospho-Regulation of the Proneural Proteins Ascl1 and Ngn2. OSF. 2018 http://www.doi.org/10.17605/OSF.IO/73NE5 10.12688/wellcomeopenres.14848.1 PMC6206610 30430141

