# Supplementary material for: Interaction between opposing modes of phospho-regulation of the proneural proteins Ascl1 and Ngn2
Source: Wellcome Open Res. 2018 Oct 1;3:129. [Version 1] doi: 10.12688/wellcomeopenres.14848.1 (PMC6206610; doi:10.12688/wellcomeopenres.14848.1)
Supplement: Peer review history

# Peer review: Referee response for version 1

**DOI:** 10.21956/wellcomeopenres.16177.r34041

**Published:** 2018-10-24

**Authors:** Kristine A. Henningfeld

**Affiliations:** 1 Department of Developmental Biochemistry, Center for Nanoscale Microscopy and Molecular Physiology of the Brain (CNMPB), University of Göttingen, Göttingen, Germany

The authors have previously shown that multi-site phosphorylation is a conserved mechanism that inhibits the neuronal-inducing activity of the proneural bHLH proteins. Correspondingly, phospho-null mutations in the N- and C-termini of Neurog2 and Ascl1 cumulatively increases the stability and proneural activity of these proteins. More recently (Quan et al., 2016), it has been shown that the proneural activity of Scute and Atonal in the *Drosophila *wing disc, as well as Neurog2 in the developing mouse cortex can be inactivated by phosphorylation of a single conserved residue. This conserved residue is located in the bHLH domain and its phosphorylation disrupts DNA binding.

 In this short research note, Hardwick and Philpott investigated which of the afore described bHLH protein phosphorylation mechanisms is dominant in the control of the proneural activity. The ability of the wild-type Ascl1 and Neurog2 to promote neuronal differentiation in comparison to versions of the proteins harboring phospho-null mutations in the N-and C-termini or a phospho-mimetic mutation in the bHLH domain alone, as well as in combination were studied. The proneural activity was assessed in gain of function assays using *X. laevis*embryos. As shown using both qPCR analysis and whole mount in situ hybridization, the phospho-mimetic mutation in the bHLH domain of Ascl1 and Neurog2 abolished their ability to promote neuronal differentiation. The inhibitory phospho-mimetic mutation was also able to override the activating effect of the phospho-null mutations in the N- and C-termini of Ascl1. Neurog2, however, still maintained weak proneural activity.

 In general, the report is well and succinctly written, the figures are clear and represent the authors conclusions. The report is of interest as it further demonstrates the conservation of phosphorylation in the bHLH domain as a dominant regulatory mechanism to inhibit proneural protein activity. Introduction of the bHLH phospho-mimetic mutation in the Neurog2 phospho-null N- and C-termini mutant (9S-A/T149D) was not sufficient to completely disrupt neuronal differentiation induced by this mutant. These findings complement that of Quan et al., which found that the Neurog2 bHLH phospho-mimetic mutant still maintains weak DNA binding activity. However, alternative mechanisms cannot be excluded.

 It will be of interest to study the phospho-null bHLH mutation in this system to further evaluate the consequences of persistent proneural activity. 

 Minor point: The authors state that the 9S-A/T149D Neurog2 mutant maintains residual activity, which is restricted to the neural ectoderm. However, for the embryo depicting this activity (Fig 2D), it appears that the injected mRNA has not targeted the nonneural ectoderm (see b-gal staining).

I have read this submission. I believe that I have an appropriate level of expertise to confirm that it is of an acceptable scientific standard.

# Peer review: Referee response for version 1

**DOI:** 10.21956/wellcomeopenres.16177.r34031

**Published:** 2018-10-17

**Authors:** Karel Dorey

**Affiliations:** 1 Faculty of Biology Medicine and Health, University of Manchester, Manchester, UK; 2 School of Medicine, University of Manchester, Manchester, UK

In this research note, the authors attempt to reconcile two different mechanisms regulating the activity of pro-neural bHLH transcription factors. The authors have previously shown very convincingly that Neurogenin2 and Ascl1 are phosphorylated on multiple SP sites by Cyclin Dependant Kinase 2 (Cdk2), preventing their ability to drive neuronal differentiation. These phosphorylation sites are located in the N- and C-terminal regions of the proteins, but not in the DNA binding domain. Furthermore, they have previously shown that the multiple phosphorylation sites can act as a “rheostat” to regulate Ngn2/Ascl1 activity. In contrast, studies from Drosophila (atonal in the retina) and in the mouse cortex (Ngn2) pinpoints to an on/off mechanism by a single phosphorylation event in the DNA binding domain. Interestingly, this phosphorylation site is very well conserved in other pro-neuronal bHLH transcription factors such as  Ascl1. The authors have therefore decided to test the possibility that both mechanisms (rheostat and on/off switch) might co-exist to regulate the activity of pro-neural bHLH in vertebrates. 

 Overall the manuscript is very well written, the methods are well established in the lab and the assays used are very robust. The data support the conclusions of the study, i.e. that both mechanisms of regulation co-exist. However, I have one major issue that the authors could address easily to improve the validity of their conclusions. The statistical analyses of their data should be improved by doing the following:

 - mean and standard deviation (which is more appropriated than standard error of the mean) should be calculated on at least three repeats rather than two. 

 - within an experiment where multiple conditions are compared (ie Figure 1c or 2b), a two-way ANOVA with post-hoc test should be applied rather than multiple t-tests. 

 - when categorising data (Fig1D/2C), a X ^2 ^should be performed.

 Given the clear differences, I am sure that these analyses will not change the conclusion of the study but there is rightly a drive for more rigorous statistical analyses of the data that are reported. 

 One minor point that could be discussed by the authors, and maybe the subject of further studies: I would have expected the S150D / T149D mutants to have a dominant negative effect (i.e. less neurogenesis) but it seems that they are inert. Is it because they do not dimerise with Xenopus Ascl1 / Ngn2? Would the authors expect the same results should they use the Xenopus orthologues?

I have read this submission. I believe that I have an appropriate level of expertise to confirm that it is of an acceptable scientific standard.
